# A national survey of the chemotherapy regimens used to treat small cell lung cancer (SCLC) in the United Kingdom

**DOI:** 10.1054/bjoc.2001.1817

**Published:** 2001-06

**Authors:** R J Sambrook, D J Girling

**Affiliations:** Medical Research Council Clinical Trials Unit, 222 Euston Road, London NW1 2DA

**Keywords:** small cell lung cancer, chemotherapy, national survey

## Abstract

Many chemotherapy regimens are used for treating SCLC in the United Kingdom, but it is not known, in any detail, which regimens are used, by which specialists, for which types of patient. We conducted a survey among all medical and clinical oncologists, respiratory physicians and general physicians with respiratory interest in the United Kingdom to find out. The questionnaire asked for the number of SCLC patients treated annually; how many were given chemotherapy; the drugs, doses and schedules chosen according to prognostic group (as defined by the clinician); and the reasons for choice of regimen. 1214 questionnaires were sent out, and responses were received from 1070 (88%) clinicians; 266 (25%) of these treated SCLC with chemotherapy. Of 4674 patients given chemotherapy annually, 36% were given it by clinical oncologists, 30% by medical oncologists, 27% by respiratory physicians, and 7% by general physicians. In all, 34 regimens were reported with 151 different combinations of dose and schedule. In 2311 good prognosis patients, 23 regimens were used, the commonest being ACE (doxorubicin, cyclophosphamide, etoposide), ICbE (ifosfamide, carboplatin, etoposide), CAV (cyclophosphamide, doxorubicin, vincristine), CbE (carboplatin, etoposide), and PE (cisplatin, etoposide). In 1517 poor prognosis patients, 21 regimens were used, the commonest being CAV, EV (etoposide, vincristine), CbE, CAV alternating with PE, and oral etoposide. 452 patients were treated regardless of prognosis and for 219 no prognostic criteria were specified. The remaining 175 were given second-line chemotherapy or were given regimens chosen to avoid toxicity or because of intercurrent disease or other reasons. The main reasons affecting choice of regimen were routine local practice, patients' convenience, quality of life considerations, trial results and cost. The results show wide variation in routine practice and will be useful in reporting and planning clinical trials and in deciding on local treatment policies. © 2001 Cancer Research Campaign http://www.bjcancer.com


British Journal of Cancer (2001) 84(11), 1447–1452
© 2001 Cancer Research Campaign
doi: 10.1054/ bjoc.2001.1817, available online at http://www.idealibrary.com on IDEAL®

http://www.bjcancer.com

### A national survey of the chemotherapy regimens used to treat small cell lung cancer (SCLC) in the United Kingdom

**RJ Sambrook and DJ Girling**

Medical Research Council Clinical Trials Unit, 222 Euston Road, London NW1 2DA

**Summary** Many chemotherapy regimens are used for treating SCLC in the United Kingdom, but it is not known, in any detail, which regimens are used, by which specialists, for which types of patient. We conducted a survey among all medical and clinical oncologists, respiratory physicians and general physicians with respiratory interest in the United Kingdom to find out. The questionnaire asked for the number of SCLC patients treated annually; how many were given chemotherapy; the drugs, doses and schedules chosen according to prognostic group (as defined by the clinician); and the reasons for choice of regimen. 1214 questionnaires were sent out, and responses were received from 1070 (88%) clinicians; 266 (25%) of these treated SCLC with chemotherapy. Of 4674 patients given chemotherapy annually, 36% were given it by clinical oncologists, 30% by medical oncologists, 27% by respiratory physicians, and 7% by general physicians. In all, 34 regimens were reported with 151 different combinations of dose and schedule. In 2311 good prognosis patients, 23 regimens were used, the commonest being ACE (doxorubicin, cyclophosphamide, etoposide), ICbE (ifosfamide, carboplatin, etoposide), CAV (cyclophosphamide, doxorubicin, vincristine), CbE (carboplatin, etoposide), and PE (cisplatin, etoposide). In 1517 poor prognosis patients, 21 regimens were used, the commonest being CAV, EV (etoposide, vincristine), CbE, CAV alternating with PE, and oral etoposide. 452 patients were treated regardless of prognosis and for 219 no prognostic criteria were specified. The remaining 175 were given second-line chemotherapy or were given regimens chosen to avoid toxicity or because of intercurrent disease or other reasons. The main reasons affecting choice of regimen were routine local practice, patients' convenience, quality of life considerations, trial results and cost. The results show wide variation in routine practice and will be useful in reporting and planning clinical trials and in deciding on local treatment policies. © 2001 Cancer Research Campaign http://www.bjcancer.com

**Keywords**: small cell lung cancer; chemotherapy; national survey

Small cell lung cancer (SCLC) responds well to combination chemotherapy regimens, but although many drugs have been shown to be active against SCLC, there is no firm evidence that one particular combination of active drugs is superior to any other (Murray, 1997) and many different chemotherapy regimens are in routine use (Ettinger, 1995). In North America, the combination of cisplatin and etoposide (PE), owing to its lack of severe non-haematological toxicity, its ease of administration, and the presumed synergy between the 2 drugs, is said to be widely used as standard for good prognosis patients (Johnson, 1993), whereas in Europe, doxorubicin, cyclophosphamide and etoposide (ACE) is said to be favoured over PE (Groen et al, 1999). It is not known with any precision, however, which policies are in routine use in the United Kingdom or which specialists are most commonly involved in administering chemotherapy to patients with SCLC.

A survey of clinicians treating lung cancer was therefore undertaken to establish which chemotherapy regimens are being prescribed, by whom, and for which prognostic groups of patient. The findings of this survey would demonstrate whether any consensus existed on standard chemotherapy and should be useful in selecting relevant control groups for randomized trials in the treatment of SCLC and in deciding local treatment policies.

*Received 17 October 2000*
*Revised 19 January 2001*
*Accepted 9 February 2001*

*Correspondence to:* DJ Girling

### METHODS

### Target population

The target population of medical and clinical (radiation) oncologists, respiratory physicians and general physicians with a special interest in respiratory medicine was identified from the Royal College of Physicians' membership list and from the General Medical Council's Specialist Register. The questionnaire was sent out in August 1998 with a covering letter explaining its aims and rationale and a stamped addressed envelope. A duplicate questionnaire was sent in January 1999 to non-responders. Those not replying to this mailing were followed up with a telephone call in March 1999, which established all those who did not treat SCLC with chemotherapy. A final mailing was sent in April 1999 to non-responders and included an additional 64 clinicians who had been identified from returned questionnaires and from our MRC lung cancer mailing list.

### The survey

The survey asked the following questions.

#### *Part 1: General questions*

Is your status consultant, professor or senior lecturer? If yes please complete and return the enclosed survey; if no please return the survey uncompleted. What is your speciality? How many newly diagnosed SCLC patients do **you treat** annually? How many of these do you treat with chemotherapy? How many **standard** chemotherapy regimens do you use for your SCLC patients *(e.g. palliative, intensive etc.)*? *Do not include regimens used in trials.*

### *Part 2: Questions about the chemotherapy regimens*

For each chemotherapy regimen indicate the criteria used to define the subgroup of patients (e.g. performance status, Manchester Score, age etc.) for which it is used; reasons for this choice, and number of patients to whom this applies. Do not include patients whom you see, but refer on without treatment.

For each regimen: The drugs (please use generic drug names): Number of cycles. Interval between cycles (weeks). Dose of each drug (mg/m$^2$). Subgroup of patients (e.g. performance status, age, extent of disease etc.) Reasons (please tick one or more): patient convenience (toxicity:efficacy ratio); trial results (which trial(s)?); cost; quality of life benefits; standard local routine protocol; other (specify). In addition there was a space for comments.

No information was requested about thoracic radiotherapy or prophylactic cranial irradiation.

**Table 1** Acronyms for the chemotherapy regimens

| Acronym | Drugs |
|---|---|
| **Platinum-based regimens** | |
| CbE | Carboplatin, etoposide |
| CbEV | Carboplatin, etoposide, vincristine |
| ChEP | Chlorambucil, etoposide, cisplatin |
| GP | Gemcitabine, cisplatin |
| ICbE | Ifosfamide, carboplatin, etoposide |
| IPE | Ifosfamide, cisplatin, etoposide |
| MIP | Mitomycin, ifosfamide, cisplatin |
| MVbP | Mitomycin, vinblastine, cisplatin |
| NP | Vinorelbine, cisplatin |
| PE | Cisplatin, etoposide |
| PET | Cisplatin, etoposide, paclitaxel |
| VICbE | Vincristine, ifosfamide, carboplatin, etoposide |
| **Doxorubicin-based regimens** | |
| ACE | Doxorubicin, cyclophosphamide, etoposide |
| AVE | Doxorubicin, vincristine, etoposide |
| AVI | Doxorubicin, vincristine, ifosfamide |
| CAV | Cyclophosphamide, doxorubicin, vincristine |
| CAVb | Cyclophosphamide, doxorubicin, vinblastine |
| CAV/PE | Cyclophosphamide, doxorubicin, vincristine alternating with PE |
| CAVE | Cyclophosphamide, doxorubicin, vincristine, etoposide |
| **Other multi-drug regimens** | |
| CEpV | Cyclophosphamide, epirubicin, vincristine |
| CE | Cyclophosphamide, etoposide |
| CEV | Cyclophosphamide, etoposide, vincristine |
| ECMxV | Etoposide, cyclophosphamide, methotrexate, vincristine |
| ChPrPdE | Chlorambucil, procarbazine, prednisolone, etoposide |
| CVLMx | Cyclophosphamide, vincristine, lomustine, methotrexate |
| EpEC | Epirubicin, etoposide, cyclophosphamide |
| EV | Etoposide, vincristine |
| EVEp | Etoposide, vincristine, epirubicin |
| VIE | Vincristine, ifosfamide, etoposide |
| VnE | Vindesine, etoposide |
| **Single-drug regimens** | |
| C | Cyclophosphamide |
| Oral E | Oral etoposide |
| Ep | Epirubicin |
| Cb | Carboplatin |

### Measures of performance status and prognosis

The WHO performance status (World Health Organization, 1979) or the prognostic scoring system, the Manchester score (Cerny et al, 1987), was used by some respondents or was derived from their responses. The WHO grades are: 0 = able to carry out all normal activity without restriction; 1 = restricted in physically strenuous activity but ambulatory and able to carry out light work; 2 = ambulatory and capable of all self-care but unable to carry out any work; up and about more than 50% of waking hours; 3 = capable of only limited self-care; confined to bed or chair more than 50% of waking hours; 4 = completely disabled; cannot carry out any self-care; totally confined to bed or chair. The Manchester score is derived from pretreatment factors as follows: 0 = starting score; +1 if lactate dehydrogenase >450 U l$^{-1}$ (upper normal limit); +1 if extensive stage; +1 if serum sodium <132 mmol l$^{-1}$; +1 if Karnofsky score <60; +1 if alkaline phosphatase >165 U l$^{-1}$ (1.5-fold upper limit); +1 if serum bicarbonate <24 mmol l$^{-1}$. The range of possible scores is 0–6.

### RESULTS

### Consultants giving chemotherapy

A total of 1214 questionnaires were sent out and responses were obtained from 1070 clinicians, giving a compliance rate of 88%. 777 of the 1070 respondents did not treat SCLC with chemotherapy; an additional 27 did give chemotherapy, but had their caseload included in the questionnaire answered by another member of their department. Of the 266 consultants who did give chemotherapy, 54 (20%) were medical oncologists, 112 (42%) clinical oncologists, 76 (29%) respiratory physicians, and 24 (9%) general physicians. Of an estimated 7724 SCLC patients treated annually, 4674 (61%) were given chemotherapy. 1695 (36%) of the 4674 given chemotherapy were treated by clinical oncologists, 1400 (30%) by medical oncologists, 1262 (27%) by respiratory physicians, and 317 (7%) by general physicians. Overall, 34 different regimens were used, with a total of 151 different combinations of dose and schedule. The acronyms of all the regimens are listed in Table 1.

### Prognostic groups

Patients were placed into broad prognostic groups on the basis of the criteria clinicians used to select chemotherapy regimens. These groups are shown in Table 2, along with the number of patients included in each group. There is inevitably a slight overlap between some of the groups (e.g. the good and the poor prognostic groups in patients with a WHO performance status of 2) because prognostic groups were not defined in the survey but left to respondents. Moreover, some clinicians chose treatment purely on the basis of extent of disease, with no reference to performance status or other prognostic factors, and some did not assign patients to prognostic groups at all or did not specify any prognostic criteria. The good performance status group includes all patients with a Manchester score of 0 or 1, whatever the extent of their disease. The poor performance status group encompasses patients with a Manchester score of 2 or more, whatever the extent of their disease. The miscellaneous groups were mainly accounted for by patients with co-morbidity. The other groups are self-explanatory, although it should be noted that we did not ask for information on second-line treatment.

 *© 2001 Cancer Research Campaign*

**Table 2** Prognostic groups derived from the survey responses and number of patients in each group

| Prognostic group[a] | Patients No. | Patients % |
|---|---|---|
| Good PS (WHO 0–2), any stage, MS 0–1 | 2262 | 48 |
| Limited disease, not otherwise defined | 49 | 1 |
| Good PS but with cardiac problems | 17 | <1 |
| Poor PS (WHO 2–4), any stage, MS 2–6 | 1406 | 30 |
| Extensive disease, not otherwise defined | 111 | 2 |
| Poor PS with cardiac problems | 30 | 1 |
| All patients except very fit | 118 | 3 |
| Ungrouped for prognosis | 334 | 7 |
| Second-line chemotherapy for relapse | 58 | 1 |
| Other miscellaneous groups | 70 | 1 |
| No prognostic criteria specified | 219 | 5 |
| Total patients | 4674 | 100 |

[a]PS = performance status; MS = Manchester score.

**Table 3** Commonest of 23 regimens prescribed for 2262 patients with good performance status and 49 with limited disease

| Regimen[a] | Patients No. | Patients % |
|---|---|---|
| ACE | 640 | 28 |
| ICbE | 288 | 13 |
| CAV | 283 | 12 |
| CbE | 277 | 12 |
| PE | 275 | 12 |
| CAV/PE | 197 | 9 |
| CAVE | 183 | 8 |
| VICbE | 36 | 2 |
| ECMxV | 23 | 1 |
| Other regimens[b] | 109 | 5 |
| Total patients | 2311 | 100 |

[a]For definitions, see Table 1.[b]Other regimens, each accounting for less than 1% of patients were, in order of frequency, MIP, AVE, MVbP, NP, AVI, E, IPE, CbEV, EpEC, CEpV, CE, CEV, EV, and PET.

### Regimens prescribed for patients with good prognosis

Table 3 shows the regimens prescribed for patients with good performance status and for those classified solely on the basis of their having limited disease. Both these groups were regarded as having relatively good prognosis and we have therefore combined them in the table. 23 regimens were reported; those most frequently used are shown, ranked, in the table. Of the 9 most frequently prescribed, 4 were platinum-based (ICbE, CbE, PE, VICbE), 4 doxorubicin-based (ACE, CAV, CAV/PE, CAVE; CAV/PE also containing cisplatin), and the ninth (ECMxV) contained neither platinum nor doxorubicin.

There was substantial variation in the drug dosages used. For example, in the ACE regimen, the most commonly prescribed regimen, the dose of doxorubicin ranged from 30–60 mg $m^{-2}$, the dose of cyclophosphamide from 600–1000 mg $m^{-2}$, and the dose of etoposide from 100–150 mg $m^{-2}$ i.v. The most frequently used combination of doses was doxorubicin 40–60 mg $m^{-2}$, cyclophosphamide 1000 mg $m^{-2}$, and i.v. etoposide 120 mg $m^{-2}$. Similar variation was seen in the other regimens. The number of cycles of chemotherapy prescribed for this group of patients ranged from 3–6, regardless of regimen, 6 being prescribed for 85% of the patients.

**Table 4** Commonest of 21 regimens prescribed for 1406 patients with poor performance status and 111 with extensive disease

| Regimen[a] | Patients No. | Patients % |
|---|---|---|
| CAV | 544 | 36 |
| EV | 264 | 17 |
| CbE | 137 | 9 |
| CAV/PE | 136 | 9 |
| Oral E | 132 | 9 |
| CAVE | 88 | 6 |
| ACE | 50 | 3 |
| CE | 41 | 3 |
| Cb | 29 | 2 |
| VIE | 25 | 2 |
| Other regimens[b] | 71 | 5 |
| Total patients | 1517 | 100 |

[a]For definitions, see Table 1.[b]Other regimens, each accounting for less than 1% of patients were, in order of frequency, ChEP, EpEC, Ep, EVEp, PE, CEpV, MVbP, AVI, CbEV, CEV, and ECMxV.

### Regimens prescribed for patients with poor prognosis

Table 4 shows the regimens prescribed for patients with poor performance status and for those classified solely on the basis of their having extensive disease. Both these groups were regarded as having relatively poor prognosis and we have therefore combined them in the table. 21 regimens were reported; the most frequently used are shown, ranked, in the table. Of the 10 most frequently prescribed, 2 were platinum-based (CbE, Cb), 4 doxorubicin-based (CAV, CAV/PE, CAVE, ACE; CAV/PE also containing cisplatin), and 4 contained neither platinum nor doxorubicin (EV, Oral E, CE, VIE). Two were single-drug regimens (Oral E, Cb).

There was substantial variation in drug dosages. In the CAV regimen, the most frequently prescribed regimen, the dose of cyclophosphamide ranged from 500–1000 mg $m^{-2}$, the dose of doxorubicin from 35–60 mg $m^{-2}$ and the dose of vincristine from 1–2 mg $m^{-2}$. The most frequently used combination of doses was cyclophosphamide 750 mg $m^{-2}$, doxorubicin 40 mg $m^{-2}$ and vincristine 1.3 mg $m^{-2}$. Similar variation was seen in the other regimens. The number of cycles of chemotherapy prescribed for this group of patients ranged from 2–6, regardless of regimen, 6 being prescribed for 61% of the patients and 4 for 31%.

### Regimens prescribed for other patient groups

Among the other groups of patients (Table 2), the distinguishing feature of regimens chosen for the 47 patients with cardiac problems (17 with good performance status, 30 with poor performance status), was that they did not contain doxorubicin; the commonest regimens were CAV, CbE, EV and PE. For the 118 patients classified as including all except the very fit, and the 334 who were not grouped by prognosis, the commonest regimens were CAV, CbE, CAVE and CAV/PE. The commonest regimens for the 70 patients in miscellaneous groups were CbE, CAV and E. Information on second-line regimens was not requested and so data on these will be incomplete.

*© 2001 Cancer Research Campaign*

### Single-drug chemotherapy

Single-drug chemotherapy was given to a total of 228 patients (5% of all patients given chemotherapy). Of these 228 patients, 166 received oral etoposide, 51 carboplatin, 10 epirubicin and 1 cyclophosphamide. Single-drug chemotherapy was given almost exclusively to poor performance status patients, for urgent palliation, or as second-line treatment.

### Reasons for choice of regimen

The reasons consultants gave for their choice of regimens (Table 5) varied according to regimen. The table shows for the 5 most commonly prescribed regimens in each of the good and poor prognosis groups, the number of consultants using each regimen, the number of patients affected, and the percentage of consultants influenced by each reason for choice of regimen.

For patients with good prognosis (upper part of table), treatment according to a standard local routine protocol was the single most frequently stated reason, but patient convenience also influenced choice substantially, particularly for CAV and CbE. Trial results were influential, especially in the choice of ICbE. Quality of life benefits had some influence for all 5 regimens; cost was the least frequently quoted reason for choice of regimen.

For patients with poor prognosis (lower part of the table), CAV, EV and Oral E were used by substantially more consultants than were CbE and CAV/PE. Treatment according to a standard local routine protocol and patient convenience were again important reasons. Patient convenience was influential for 9 of the 10 consultants prescribing CbE, as were quality of life benefits. Cost was, again, the least frequently reported reason for choice of regimen.

### DISCUSSION

The high level of compliance by clinicians in responding to this survey suggests that they considered the questions clinically important and the findings potentially interesting. The reliability of the results of such surveys is bound to depend, in part, on the compliance rates achieved. If a survey is perceived as addressing important issues, good compliance should be the aim and should be expectantly pursued. It is essential to explain to those surveyed the reasons for conducting a survey and why the findings are likely to prove important.

In the present survey, clinical (i.e. radiation) oncologists were the specialists most involved in giving chemotherapy. The others, in order of the number of patients treated with chemotherapy, were medical oncologists, respiratory physicians, and general physicians with a special interest in respiratory disease. The major involvement of clinical oncologists can almost certainly be explained by the small number of medical oncologists in this country and the large number of patients requiring treatment. It is important that all specialists giving chemotherapy are trained to do so, and have available the necessary oncology trained staff and facilities. According to the recently published Guidance on Commissioning Cancer Services: Improving Outcomes in Lung Cancer (NHS Executive, 1998), chemotherapy should be given in units or centres where close supervision by oncologists and chemotherapy nurse specialists, expert pharmacy and 24-hour laboratory support are available. Patients receiving chemotherapy should have access to emergency care, information and advice from oncology trained staff on a 24-hour basis. It is particularly important to ensure that these requirements are met when chemotherapy is being given within a number of different clinical specialities.

A wide variety of chemotherapy regimens is being used to treat small cell lung cancer. This variety is partly to be expected because different regimens are appropriate for different groups of patients. The fittest are typically those with good performance status, limited disease, no major symptoms, little if any co-morbidity, and able to tolerate intensive chemotherapy and high-dose thoracic radiotherapy given with curative intent. At the other end of the range are people with poor performance status, extensive disease, major symptoms, major co-morbidity, and only able to tolerate relatively non-toxic, palliative treatment aimed at controlling their symptoms, improving their quality of life, prolonging survival and keeping them at home as much as

**Table 5** Reasons given by consultants for choice of the most commonly prescribed regimens

| **Patients with good prognosis** **Regimen** | **ACE** | **ICbE** | **CAV** | **CbE** | **PE** |
|---|---|---|---|---|---|
| Number of consultants | 60 | 35 | 26 | 26 | 31 |
| Number of patients | 640 | 288 | 283 | 277 | 275 |
| Reason for choice (% consultants) | | | | | |
| Patient convenience | 43% | 29% | 81% | 69% | 29% |
| Trial results | 32% | 71% | 50% | 23% | 26% |
| Cost | 18% | 0% | 46% | 8% | 14% |
| Quality of life benefits | 25% | 37% | 46% | 43% | 29% |
| Standard local routine protocol | 77% | 57% | 69% | 73% | 57% |
| **Patients with poor prognosis** **Regimen** | **CAV** | **EV** | **CbE** | **CAV/PE** | **Oral E** |
| Number of consultants | 63 | 36 | 10 | 10 | 37 |
| Number of patients | 544 | 264 | 137 | 136 | 132 |
| Reason for choice (% consultants) | | | | | |
| Patient convenience | 49% | 67% | 90% | 30% | 57% |
| Trial results | 43% | 69% | 10% | 50% | 3% |
| Cost | 24% | 19% | 10% | 20% | 5% |
| Quality of life benefits | 37% | 56% | 90% | 20% | 57% |
| Standard local routine protocol | 60% | 61% | 60% | 60% | 41% |

© 2001 Cancer Research Campaign possible. The survey has, however, revealed great variety within prognostic groups of patients, suggesting much uncertainty about which regimens are considered to confer the greatest benefits in terms of survival and quality of life. There is clearly no general agreement that one chemotherapy regimen, or one of a small group of regimens, should be regarded as standard treatment.

This multiplicity of standard regimens has come about because no large randomized trial has confirmed the superiority of one over any other of as many as a dozen different combination chemotherapy regimens. This is reflected in the above-mentioned NHS Executive document which states that no regimen has been shown to be clearly superior to a variety of combinations (usually between 2 and 4) of the following drugs: cyclophosphamide, doxorubicin, vincristine, etoposide, cisplatin/carboplatin, ifosfamide.

In all, 23 regimens were used to treat patients with relatively good prognosis. The 9 most commonly used regimens accounted for 95% of these patients, but they varied greatly in the drugs they contained, and platinum drugs were clearly not considered essential. In currently open randomized trials in the United Kingdom, regimens in use as standard control regimens include ACE and PE by the Medical Research Council, and CAV/PE by the London Lung Cancer Group. In North America, PE is widely regarded as standard (Murray 1997), while ACE is considered standard by the European Organization for Research and Treatment of Cancer (Groen et al, 1999). The Textbook of Lung Cancer recently published by the International Association for the Study of Lung Cancer (IASLC) lists the following regimens as commonly used and, by implication, standard: PE, CAV, ACE, and CbE (Carney and Shepherd, 2000).

For patients with poor prognosis, 21 regimens were used, the 10 most frequently used accounting for 95% of the patients. Among these 10, 5 (CAV, CbE, CAV/PE, CAVE, ACE) were also among the 9 most commonly used to treat patients with good prognosis. There still seems to be a role for some single agents which have been shown to be inferior to multi-drug regimens, in terms of quality of life or survival or both. Oral etoposide on its own, for example, was used in 9% of patients with poor performance status, despite the proven superiority of intravenous combinations (CAV or EV) and the higher risk of haematological toxicity with oral etoposide in this group of patients (Medical Research Council Lung Cancer Working Party, 1996; Souhami et al, 1997). Both the NHS Executive document and the IASLC textbook recommend combination chemotherapy in this group of patients.

The most commonly used regimens: ACE, ICbE, CAV and Cb(or P)E for patients with relatively good prognosis, and CAV, EV, CbE and CAV/PE for patients with poor prognosis, seem reasonable choices. There was wide variation, however, in the dosages prescribed. Such variation is of particular concern in patients with relatively good prognosis, in whom an important aim of treatment is to prolong survival and achieve cure in some patients. Arriagada and colleagues (1993) found that giving higher doses of cyclophosphamide and cisplatin for just the first cycle of a 6-cycle regimen improved survival. The best dosages in terms of survival benefits and acceptable toxicity need to be defined for commonly used regimens so that the best outcomes can be achieved in routine practice.

The most commonly used regimens included both platinum-based and doxorubicin-based drug combinations. A meta-analysis of published data from 4054 evaluable patients in 19 randomized trials comparing a cisplatin-containing regimen versus a non-platinum regimen investigated survival, response and toxicity (Pujol et al, 2000). It showed that a cisplatin-containing regimen yielded a higher response rate and probability of survival than did a regimen containing other alkylating agents without a perceptible increase in risk of toxic death. No direct comparison was made, however, between platinum-based and doxorubicin-based regimens. Further research is needed to determine the most active and most acceptable drug combinations in the treatment of small cell lung cancer.

The great majority of patients with relatively good prognosis were prescribed 6 cycles of chemotherapy, and of those with poor prognosis 4 or 6 cycles. This is in keeping with findings from a number of randomized trials that investigated duration of chemotherapy (Cullen et al, 1986; Medical Research Council Lung Cancer Working Party, 1989, 1993; Spiro et al, 1989; Giaconne et al, 1993).

A Medical Research Council Lung Cancer Working Party trial has shown that in the treatment of patients with good performance status, increasing the dose-intensity of ACE chemotherapy by means of granulocyte colony-stimulating factor support improved survival while maintaining acceptable toxicity (Thatcher et al, 2000). Further research is needed to establish whether similar improvements can be achieved with other regimens, or whether other strategies, such as concurrent multiple-daily-fraction radiotherapy and PE chemotherapy (Turrisi et al, 1999) would be preferable.

### ACKNOWLEDGEMENT

The authors thank all the clinicians who collaborated in this survey, thereby providing a very high compliance rate and correspondingly reliable data.

### REFERENCES

Arriagada R, Le Chevalier T, Pignon J-P, Rivière A, Monnet I, Chomy P, Tuchais C, Tarayre M and Ruffié P (1993) Initial chemotherapeutic doses and survival in patients with limited small-cell lung cancer. *N Engl J Med* **329**: 1848–1852

Carney DN and Shepherd FA (2000) Treatment of SCLC: Chemotherapy. In: *Textbook of Lung Cancer*, Hansen HH (ed) pp 261–272. Martin Dunitz: London

Cerny T, Blair V, Anderson H, Bramwell V and Thatcher N (1987) Pretreatment prognostic factors and scoring system in 407 small cell lung cancer patients. *Int J Cancer* **39**: 146–149

Cullen M, Morgan D, Gregory W, Robinson M, Cox D, McGivern D, Ward M, Richards M, Stableforth D, MacFarlane J, Fletcher J, David D and the Midlands Small Cell Lung Cancer Group (1986) Maintenance chemotherapy for anaplastic small cell carcinoma of the bronchus: a randomised, controlled trial. *Cancer Chemother Pharmacol* **17**: 157–160

Ettinger DS (1995) New drugs for treating small cell lung cancer. *Lung Cancer* **12** (supplement 3): S53–S61

Giaconne G, Delasio O, McVie GJ, Kirkpatrick A, Postmus PE, Burghouts JTM, Bakker W, Koolen MGJ, Venrik CPJ, Roozendaal KJ, Planting AST, van Zandwijk N, ten Velde GJM, Splinter TAW, for the European Organization for Research and Treatment of Cancer Lung Cancer Cooperative Group (1993) Maintenance chemotherapy in small-cell lung cancer: long-term results of a randomized trial. *J Clin Oncol* **11**: 1230–1240

Groen HJM, Fokkema E, Biesma B, Kwa B, Putte NJWG van, Postmus PE and Smit EF (1999) Paclitaxel and carboplatin in the treatment of small-cell lung cancer patients resistant to cyclophosphamide, doxorubicin, and etoposide: a non-cross-resistant schedule. *J Clin Oncol* **17**: 927–932

Johnson DH (1993) Treatment of limited-stage small cell lung cancer: recent progress and future directions. *Lung Cancer* **9** (supplement 1): S1–S19

Medical Research Council Lung Cancer Working Party (1989) Controlled trial of twelve versus six courses of chemotherapy in the treatment of small-cell lung cancer. *Br J Cancer* **59**: 584–590

Medical Research Council Lung Cancer Working Party (1993) A randomised trial of three or six courses of etoposide, cyclophosphamide, methotrexate and vincristine or six courses of etoposide and ifosfamide in small cell lung cancer (SCLC) I: survival and prognostic factors. *Br J Cancer* **68**: 1150–1156

© 2001 Cancer Research Campaign 

Medical Research Council Lung Cancer Working Party (1996) Comparison of oral etoposide and standard intravenous multidrug chemotherapy for small-cell lung cancer: a stopped multicentre randomised trial. *Lancet* **348**: 563–566
Murray N (1997) Treatment of small cell lung cancer: the state of the art. *Lung Cancer* **17** (supplement 1): S75–S90
NHS Executive (1998) *Guidance on Commissioning Cancer Services; Improving Outcomes in Lung Cancer, The Research Evidence*. Department of Health: London
Pujol J-L, Carestia and Daurès J-P (2000) Is there a case for cisplatin in the treatment of small-cell lung cancer? A meta-analysis of randomized trials of a cisplatin-containing regimen versus a regimen without this alkylating agent. *Br J Cancer* **83**: 8–15
Souhami RL, Spiro SG, Rudd RM, Ruiz de Elvira MC, James LE, Gower NH, Lamont A and Harper PG (1997) Five-day oral etoposide treatment for advanced small-cell lung cancer: randomized comparison with intravenous chemotherapy. *J Natl Cancer Inst* **89**: 577–580
Spiro SG, Souhami RL, Geddes DM, Ash CM, Quinn H, Harper PG, Tobias JS, Partridge M and Eraut D (1989) Duration of chemotherapy in small cell lung cancer: a Cancer Research Campaign trial. *Br J Cancer* **59**: 578–583
Thatcher N, Girling DJ, Hopwood P, Sambrook RJ, Qian W, Stephens RJ, for the Medical Research Council Lung Cancer Working Party (2000) Improving survival without reducing quality of life in small-cell lung cancer patients by increasing the dose-intensity of chemotherapy with granulocyte colony-stimulating factor support: results of a British Medical Research Council multicentre randomized trial. *J Clin Oncol* **18**: 395–404
Turrisi AT III, Kim K, Blum R, Sause WT, Livingston RB, Komaki R, Wagner H, Aisner S and Johnson DH (1999) Twice-daily compared with once-daily thoracic radiotherapy in limited small-cell lung cancer treated concurrently with cisplatin and etoposide. *N Engl J Med* **340**: 265–271
World Health Organization (1979) *WHO Handbook for Reporting Results of Cancer Treatment. WHO offset publication no. 48*. WHO: Geneva.

*© 2001 Cancer Research Campaign*
